# Using Touchscreen Electronic Medical Record Systems to Support and Monitor National Scale-Up of Antiretroviral Therapy in Malawi

**DOI:** 10.1371/journal.pmed.1000319

**Published:** 2010-08-10

**Authors:** Gerald P. Douglas, Oliver J. Gadabu, Sabine Joukes, Soyapi Mumba, Michael V. McKay, Anne Ben-Smith, Andreas Jahn, Erik J. Schouten, Zach Landis Lewis, Joep J. van Oosterhout, Theresa J. Allain, Rony Zachariah, Selma D. Berger, Anthony D. Harries, Frank Chimbwandira

**Affiliations:** 1 Baobab Health Trust, Lilongwe, Malawi; 2 Department of Biomedical Informatics, University of Pittsburgh, Pittsburgh, Pennsylvania, United States of America; 3 Maame Akua, Area 43, Lilongwe, Malawi; 4 Department for HIV and AIDS, Ministry of Health, Lilongwe, Malawi; 5 International Training and Education Center for Health, Seattle, Washington, United States of America; 6 Management Sciences for Health, Lilongwe, Malawi; 7 Department of Medicine, College of Medicine, University of Malawi, Blantyre, Malawi; 8 Operational Research Unit, Medecins sans Frontieres, Luxembourg, Luxembourg; 9 International Union against Tuberculosis and Lung Disease, Paris, France; 10 London School of Hygiene and Tropical Medicine, London, United Kingdom

## Abstract

Gerry Douglas and colleagues describe the rationale and their experience with scaling up electronic health records in six antiretroviral treatment sites in Malawi.

## Background

The scale-up of antiretroviral therapy (ART) in sub-Saharan Africa is unprecedented. Effective monitoring and evaluation (M&E) systems are essential to track patient access to and retention on ART; to encourage feedback to improve clinic-based care; and to ensure rational drug forecasting and timely procurement to prevent drug stock-outs [1]. Complete and accurate data are a fundamental prerequisite for any M&E system.

Standardized tools for ART data collection were in use in all 297 public and private sector ART sites in Malawi by December 2009. Most sites use a paper-based system, consisting of treatment cards for each patient and one ART patient register per clinic. Their use in case-finding and treatment follow-up have been described previously [2]. Every quarter, ART clinic personnel perform a standardized patient cohort analysis that includes aggregation of case-finding details of patients registered during the previous quarter and since ART was first begun, as well as treatment outcomes for the cumulative cohort. The latter analysis requires a review of all treatment cards in order to update the clinic register and then a tally-score on current patient outcomes. “Primary outcomes” include alive on ART, died, stopped ART, transferred to another ART clinic, and lost to follow-up. Secondary outcomes for patients alive on ART include ART regimen, drug adherence, ART side effects, and current tuberculosis status. A rolling “cohort survival analysis” is also performed by counting primary outcomes of patients who registered during specified previous quarters [2].

By December 2009, 46 ART clinics in Malawi had each registered >2,000 patients and 11 of these had registered >5,000 patients [3]. At most of these high-burden clinics, nurses and medical record clerks can take up to 5 days to prepare the quarterly cohort report, sometimes closing the clinic in order to complete this task. While the quality of the quarterly case-finding data is usually good, the completeness and accuracy of primary outcome data are often compromised, and secondary outcome analyses are usually no longer feasible due to time constraints. These challenges are not limited to Malawi: a recent study conducted in 15 countries across Africa, South America, and Asia concluded that data quality at ART treatment sites is generally unsatisfactory [4]. Lack of access to evidence-based information remains a major barrier to improving healthcare in the developing world [5]–[7].

We believe that paper-based systems cannot work efficiently or accurately once large patient numbers are reached and that computerized systems are essential, a position supported by experienced practitioners in this field [8]. Furthermore, we believe that the traditional approach to M&E where the M&E process exists as a separate layer or “wrapper” around the clinical process has several shortcomings. Recognizing that M&E data and patient care data are essentially one [8], we advocate an approach that tightly couples M&E and patient care processes, recording data once at the point-of-care (POC).

Here, we describe the rationale and experience of using a touchscreen electronic medical record (EMR) system at the POC to monitor and support ART scale-up in Malawi. The EMR eliminates the process of manual updating of paper registers and data aggregation for cohort and survival analysis reports, and produces cohort reports at the touch of a button on the screen. We present a model, developed and refined in Malawi, that has the potential to improve data quality and clinical efficiency in low-resource settings by integrating EMR and M&E systems.

## Start of ART EMR in Malawi

**Figure 1:**
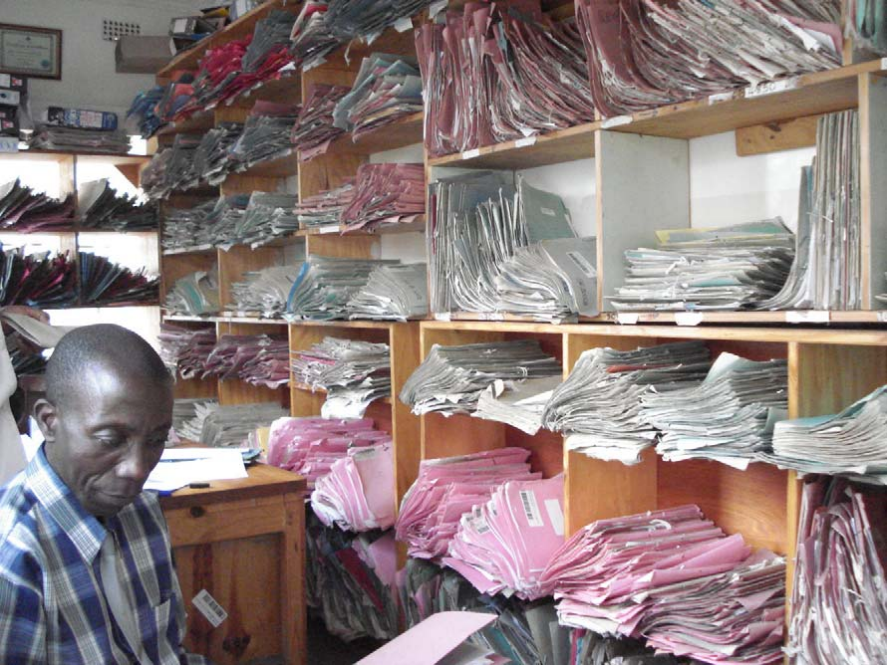
ART Clinic filing room at Queen Elizabeth Central Hospital. The man pictured in Figure 1 has given written informed consent (as outlined in the PLoS consent form) allowing the publication of his photograph. Image credit: Joep J. van Oosterhout.

In 2005, a task force created by the Department for HIV and AIDS, Ministry of Health, Malawi (MoH), investigated the feasibility of introducing computers to capture patient data and produce cohort reports at ART clinics. Two EMR operational models were considered. The first (based on several systems used elsewhere in developing countries [9]–[15]) employed a dedicated clerk to enter patient information retrospectively from patient treatment cards into a single desktop computer. The second had computers in every clinic room, connected to a central server that stored the data. With the latter model, healthcare workers would use touchscreen computers to enter patient information during clinical encounters at POC. Based on experiences of using touchscreen systems in various domains in healthcare in Malawi since 2001 [15]–[24], the task force chose the second model and established core functionality requirements for the touchscreen POC system. Pilot implementation started at the ART clinic at Queen Elizabeth Central Hospital (QECH), Blantyre, in April 2006, funded by the Global AIDS Program of the Centers for Disease Control and Prevention in Malawi. The selection of QECH was based on both high patient burden resulting in failure to manually produce quarterly reports (see Figure 1) and ease of site access. Patient treatment cards and clinic registers were maintained until there was evidence that the EMR was working reliably, had been incorporated into daily use, and was institutionalized within the clinic.

## Engineering an EMR Solution

In creating the EMR, two guiding principles directed our design choices towards balancing the needs of system users equally with other stakeholder groups. First, the EMR must improve the work of the system user, in other words, “a person working in partnership with an information resource is ‘better’ than the same person [working] unassisted”, a proposed fundamental theorem in the field of biomedical informatics [25]. Second, the EMR must ease the work of the users in compelling ways by providing a value proposition for the user, an issue that receives insufficient attention in the development of EMR systems [26].

Early work in developing an EMR system in Kenya encountered four main barriers: 1) low computer literacy among the target user group, 2) lack of unique patient identifiers, 3) difficulty in maintaining clean and reliable electrical power, and 4) managing the transition from paper to an electronic medium [10]. These problems, cited by others working in resource-constrained settings, represent core challenges to be addressed. During early pilot work in Malawi, we approached each of these challenges using hardware and software innovations [23].

First, we addressed the issue of computer literacy among the target user group, as this had important design implications for both hardware and software. We focused on a touchscreen computer with a graphical user interface as a solution that was easy to learn and use [22].

Second, we realized that the absence of unique patient identifiers (IDs) was a significant barrier to maintaining continuity of care, a fundamental requirement in the management of life-long chronic illnesses such as HIV/AIDS, diabetes mellitus, and hypertension. We chose therefore to build and deploy a system for issuing patient ID numbers. The system was first piloted at the central hospital in Malawi's capital city in March 2001. It has issued more than 650,000 nationally unique patient IDs since going live (and more than 1,100,000 IDs nationally across six sites).

Third, we discounted the idea of using uninterruptible power supplies, as they provide an inadequate length of backup time to meet the demands of multi-hour blackouts common in low-income countries. We also rejected the idea of using a generator, as this requires logistical support for procuring and topping-up fuel that we believed to be unsustainable. We favored a centralized approach that would keep the entire “system” running that has the added advantage of requiring surge protection at a single location only, where the backup system is connected to the main electrical grid. We borrowed from the telecommunications industry, which has a long history of using 48 volts DC to reliably run electronic equipment without interruption. Our solution utilizes four locally available deep-cycle 12-volt batteries of the type used in solar power installations. To further extend the duration of backup power, we utilize low-power appliance-model hardware. A complete description of the power solution is given in Text S1.

Fourth, we addressed the transition from paper to an electronic system by adopting a POC approach. Paper-based data collection tools such as patient treatment cards were replaced with touchscreen clinical workstation (TCW) appliances. Recognizing that a “paperless” system was not yet feasible, we created a “paper-enhanced” system, where information such as patient visit summaries and medication prescriptions could be printed onto inexpensive adhesive labels and affixed to existing paper artifacts such as patient-kept health passports or patient discharge forms. To facilitate this transition, a newly formatted treatment card was created upon which labels documenting the clinic visit for each patient are affixed. A summary of these and other challenges and our solutions is provided in Text S2.

Software-related design decisions included a commitment to use open-source software and selecting programming languages that local software developers could quickly develop proficiency with (Text S3).

## Use and Features of the EMR

At a typical ART clinic, healthcare workers interact with patients at multiple sequential locations or POC stages (registration, vital signs recording station, nurse review room, clinician review room, pharmacy). The software application is designed to support and enforce adherence to a standard workflow, but is sufficiently flexible to support multiple workflows (Text S4).

At each POC, healthcare workers use password-protected TCW appliances. The TCW comprises a low-power panel-PC-style touchscreen computer (no mouse or keyboard), augmented with a thermal label printer and barcode scanner. A description of the TCW and a rationale for the “information appliance” approach is provided in Text S5. Once logged in, healthcare workers initiate a session by scanning the barcoded label on the patient's health passport (Text S6). This starts the application at the correct stage for that patient.

The EMR guides the healthcare worker through a series of questions, one at a time, according to the Malawi ART guidelines. Touch-friendly screens are generated from standard HTML Web forms using the Touchscreen Toolkit (Text S7). While using the system, the healthcare worker has access to a limited past medical history through a patient dashboard and receives alerts (e.g., patient body mass index <18 – start nutritional support) and reminders (e.g., >6 months since previous CD4 lymphocyte count test). The system also performs clinical calculations (e.g., drug adherences) and facilitates medication prescribing and dispensing. Once the encounter is complete, a summary of the patient's visit is printed on an adhesive label and affixed in the patient's health passport (Text S6).

The system provides a complete set of automated reports for M&E, based on national requirements (Table 1). On-screen reports are “active”, allowing the user to tunnel down to a patient list from any indicator.

**Table 1:** Characteristics and Treatment Outcomes of ART Patients Registered at the Six EMR Sites in Malawi up to December 31, 2009.

**Characteristics of patients starting on ART**	Patients registered for ART		42,834
	Patients transferred in on ART		5,943 (14)
	Patients newly initiated on ART		36,891 (86)
	Males (%) (all ages)		17,770 (41)
	Non-pregnant females (all ages)		22,575 (53)
	Pregnant females (%) (all ages)		2,489 (6)
	Adults (%) (15 years or older at ART initiation)		39,165 (91)
	Children (%) (18 months–14 years at ART initiation)		3,241 (8)
	Infants (%) (0–17 months at ART initiation)		428 (1)
	Number (%) on ART due to:		
	Presumed severe HIV disease in infants		31 (0)
	Confirmed HIV infection in infants (PCR)		21 (0)
	WHO stage 1 or 2, CD4 below threshold		11,093 (26)
	WHO stage 2, total lymphocytes <1,200/mm^3^		0 (0)
	WHO stage 3		23,954 (56)
	WHO stage 4		7,090 (17)
	Unknown/other reason outside guidelines		645 (2)
	Number (%) on ART due to:		
	TB (%) (any form, history of TB, or current TB)		7,381 (17)
	Kaposi's sarcoma		2,120 (5)
**Treatment outcomes**	Number (%) alive and on ART		24,524 (57)
	Number (%) died		3,953 (9)
	Number (%) dying in first month		1,012 (2)
	Number (%) dying in second month		773 (2)
	Number (%) dying in third month		414 (1)
	Number (%) dying after third month		1,754 (4)
	Number (%) defaulted (>2 months after ARVs finished)		5,679 (13)
	Number (%) stopped treatment		362 (1)
	Number (%) transferred out		7,961 (19)
	Number (%) unknown outcome (pre-EMR, files lost)		355 (1)
	Of those alive and on ART:		
	1st line (start)	d4T 3TC NVP	21,943 (89)
	1st line alternatives	AZT 3TC NVP	1,377 (6)
		d4T 3TC EFV	649 (3)
		AZT 3TC EFV	150 (1)
	2nd line adult	AZT 3TC TDF LPV/r	299 (1)
	2nd line child	ddl ABC LPV/r	17 (0)
	Non-standard regimen	Any other regimen	89 (0)
	Of those alive and on ART:		
	Number (%) with side effects		1,023 (4)
	Number where pill counts were done in last month of quarter		21,284
	Number (%) with pill count in last month of quarter ≤8		21,064 (99)
	Number (%) adherent		17,368 (82)

To produce these data manually can take up to 5 days per site, and may involve clinic closure to allow staff to clean data and compile reports. Using reporting tools built into the EMR, cohort reports can be produced in just a few minutes.

ABC, abacavir; AZT, zidovudine; ddI, didanosine; D4T, stavudine; EFV, efavirenz; LPV/r, lopinavir/ritonavir; NVP, nevirapine; PCR, polymerase chain reaction; TB, tuberculosis; 3TC, lamivudine.

We address the issue of scalability, localization, and customization of the EMR in Text S8. A list of the benefits afforded by the POC model is provided in Text S9.

## Results

By December 2009, the EMR had been deployed at six ART sites in Malawi. Table 1 shows case finding and treatment outcome details of the 42,834 patients registered across the six sites.

In 2007, ten users at QECH were interviewed with a 25-question instrument to measure their impressions of the pilot EMR [27]. Seven (70%) of the users expressed a preference for the touchscreen over the paper system; two more (20%) had no preference. However, every respondent identified ongoing problems with the system that needed to be addressed and rectified as soon as possible. The detailed feedback from the survey was used in creating version 2 of the EMR. Version 2 was subsequently deployed at QECH.

In late 2007, version 2 of the EMR was piloted at two district hospitals (Dedza and Salima), and evaluated by the task force [28]. The evaluation concluded that the system met both patient care and programmatic monitoring objectives, and emphasized the importance of refining the approach of maintaining stable power, data communications, and user training. Based on the findings of this evaluation, the MoH endorsed the EMR for national use.

In 2008, a study comparing the accuracy of data captured at POC versus the same data entered retrospectively from treatment cards in the ART clinic at QECH showed that data were equally, or more, accurate when captured at POC in this setting [19],[20].

## Lessons Learned in Rolling Out the EMR in Malawi

A number of valuable lessons have been learned that will inform future roll-out of the system at other high-burden sites. We aim to have at least 50,000 ART patients managed by the EMR system by December 2010.

Producing complete and accurate cohort reports has been challenging for several reasons. First, errors, inconsistencies, and incompleteness of data in paper records must be resolved prior to back-entry of data from patient treatment cards, a process required for cumulative cohort analysis after the EMR is deployed. Second, data entry errors are made at the POC. To address this, we introduced a system to improve data validation at the POC utilizing dynamically adjusted validity ranges. Here, users are warned when data values appear implausible, in addition to disallowing values entirely when completely out of range (Text S10). A data-cleaning module has been added to the system, enabling staff to systematically identify and amend inconsistent and incomplete records. Data cleaning is now carried out monthly, significantly improving data quality. Third, there were errors in reports due to incorrect software logic, e.g., cohort reports need to be censored on specific dates, ignoring all data collected after the censor date. Identifying and working through these complexities was not straightforward and took several iterations to resolve.

Validating the accuracy of EMR data remains challenging. The MoH supervision team observed that personnel operating a paper system for a long time were more inclined to trust paper records if inconsistencies with electronic reports were seen. To allow EMR data to be more easily accessible and provide users with tools for systematic checking, we added a module to allow staff and mentors to perform systematic reviews of patient records, view graphs displaying drug adherence levels, and produce tables showing inconsistent EMR use. Initial feedback by clinic staff and supervisors has been encouraging, emphasizing that provision of EMR monitoring tools is a key requirement for successful implementation.

## Conclusions

Implementing a POC EMR has been more challenging than initially anticipated. Many of the technical difficulties have been addressed and resolved in the 8 years of ongoing system development in Malawi. However, the success of a POC system ultimately depends as much on a commitment from system users as on the technologies employed. Poor adherence to system use will result in incomplete data. Supervision is a necessary but insufficient requirement to ensure system use. Health workers will not adopt a system if they do not find sufficient value in it. Consequently, we believe that the primary challenge is to identify and address the value proposition for the user. This is an iterative process that requires a commitment to regular and ongoing dialog with the users if this paradigm shift to POC system use is to be sustainable. We believe that the experience gained, and infrastructure built, through successful deployment of the ART EMR will facilitate further roll-out to other high-burden ART sites in Malawi. We also see great potential for its adaptation and use for other chronic diseases such as tuberculosis, diabetes mellitus, and hypertension, preparing a foundation for a comprehensive electronic health record system.

## Supporting Information

Text S1 Power Backup Solutions and Low-Power Computing
(0.17 MB PDF)

Text S2 Challenges and Solutions in Implementing the EMR in Malawi
(0.01 MB PDF)

Text S3 Commitment to Open-Source Software
(0.10 MB PDF)

Text S4 Importance of Flexibility to Support Varying Workflows
(0.09 MB PDF)

Text S5 The Touchscreen Clinical Workstation Appliance
(0.06 MB DOC)

Text S6 The Malawi Health Passport
(0.12 MB PDF)

Text S7 The Touchscreen Toolkit
(0.53 MB PDF)

Text S8 Scalability, Localization, and Customization of the ART EMR
(0.01 MB PDF)

Text S9 Benefits of the Point-of-Care Model
(0.01 MB PDF)

Text S10 Dynamic and Two-Level Data Validation
(0.07 MB PDF)
