# Supplementary material for: Using Touchscreen Electronic Medical Record Systems to Support and Monitor National Scale-Up of Antiretroviral Therapy in Malawi
Source: PLoS Med. 2010 Aug 10;7(8):e1000319. doi: 10.1371/journal.pmed.1000319 (PMC2919419; doi:10.1371/journal.pmed.1000319)
Supplement: Text S1 — Power Backup Solutions and Low-Power Computing (0.17 MB PDF) [file pmed.1000319.s001.pdf]

Centralized Power Backup System and Low-Power Computing

A reliable power source is a core requirement for using an electronic medical record (EMR) system in resource-constrained settings. The use of the EMR at point-of-care (POC) further increases the necessity for uninterrupted power. Here we present a model built on two complimentary principles. First, the importance of a power backup system, and second, the importance of using low-power computing devices to increase the length of backup time.

Power Backup System

Power interruptions in Malawi can be frequent and prolonged, particularly during the rainy season (November to April). Conversely, load shedding, the practice of scheduled rotating blackouts to conserve power, is common during the dry season. When a system is used in real-time it must be highly available. Backup power is an essential prerequisite to keeping a system running when the supply of electrical power is unstable or unreliable. Choices for backup power are somewhat limited. Gas or diesel-powered generators can be used to provide power during a power interruption, but they have several limitations. Due to the noise when running, generators need to be placed outside a building, posing a risk of theft. They require periodic maintenance to run reliably, and require fuel to run. These are significant challenges in a resource-constrained setting. Since most generators only start once the power has failed there is a period of a few seconds when no power is available. This causes all computers to reboot, which can be disruptive, and may cause a loss of information. The use of a generator also requires a mechanism for the procurement and ongoing supply of fuel, which can be challenging.

An alternative method of keeping computers running in the event of a power interruption is the use of uninterruptible power supply (UPS). Under normal operating conditions, the mains alternating current (AC) power passes through the UPS directly to the

computer while simultaneously keeping a battery internal to the UPS on charge. In the event of a power failure the battery is used to power a small inverter inside the UPS that generates AC power to keep the computer operative. UPSs come in a variety of sizes and are rated in Watts. The appropriate size is determined by the amount of power the computer (or other device) consumes and the desired run time after the mains power has failed. A 350-Watt UPS purchased in the United States costs in the order of $75; however they are heavy and shipping costs are expensive. The same model of UPS purchases in Malawi costs over three times as much. A UPS of this size can keep a small computer running for 30 to 45 minutes and a server class computer for 15 to 20 minutes only. Larger UPSs can be purchased to provide longer up-times, but at much greater expense, and must be imported. Additionally the local capacity to repair UPSs in Malawi is extremely limited.

Small single-purpose UPSs are powered by an internal Gel Cell battery. The cost of a 12V, 7 Amp-hour (Ah), Gel Cell battery for a UPS is approximately $25 in Malawi (~$3.58 per Ah). Storage of electricity using large deep-cycle batteries is significantly more cost-effective. Deep-cycle batteries are locally available in most developing countries from solar power solutions providers. A 12V, 100 Ah deep-cycle battery is approximately $180 (~$1.80 per Ah), or roughly half the cost per Amp-hour.

Power from deep-cycle batteries can be utilized in two ways: 1) providing energy to an inverter that turns the direct current (DC) from the battery into AC to power the computer, or 2) providing direct DC power to operate the computer directly. All computers use DC internally but have an AC input and an internal power supply that converts the energy to DC. If the AC power supply cannot easily be bypassed, then the inverter solution may be the only option. Unfortunately, this is somewhat inefficient. The inverter will have a conversion efficiency of approximately 87%. Once the AC reaches the power supply internal to the computer it is again turned back into DC with a similar degree of inefficiency. The combined

losses can reduce the capacity of the stored energy in the battery by 25% or more. This means that a set of batteries holding sufficient capacity to power a system for 10 hours will run it for 7.5 hours or less if pro-rated linearly. However, the discharge rate is non-linear, resulting in an even greater reduction in backup time.

The telecommunications industry has a number of valuable lessons to share. Telecom has standardized on –48 Volts DC in their telephone switching stations (phone lines all over the world carry –48VDC). Certain computer hardware vendors (Dell, IBM, Compaq) make special DC power supplies for some of their server-class computers to meet the needs of the telecommunications industry. Native 48VDC network switches are also available. The solar industry, originally dedicated to using 12 Volt and 24 Volts systems has now expanded to the use of 48 Volts system, thus increasing the availability of devices for charging and maintaining 48VDC systems.

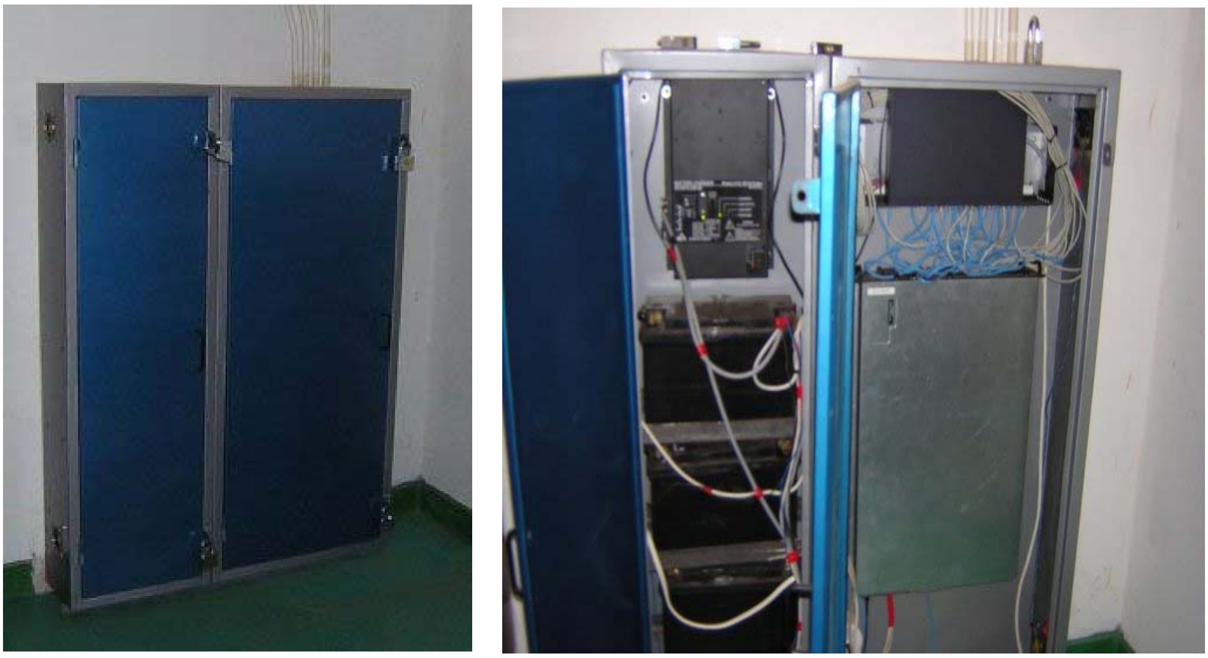

Figure S1.A: Battery backup cabinet collocated with server/network electronics cabinet

(Image Credit: Rashid Deula)

During initial pilot work we were able to procure dual redundant –48VDC power supplies for our Dell PowerEdge 2650 server, which we powered from a set of deep-cycle batteries. In a typical installation at a small site, the server and network switch are co-located in a locally manufactured steel cabinet for security and protection. In an adjacent cabinet there is a set of four 12Volt, 100 Ah deep-cycle batteries connected in series and connected to a 48Volt battery charger powered by the main grid. Figure S1.A shows an early prototype of a server and power backup cabinet installed at an HIV testing and counseling center in 2003. The four 12 Volt deep-cycle batteries, connected in series, are providing energy constantly to the server and are on charge as long as grid AC power is present to power the charger. When an interruption in grid electricity occurs, the batteries continue to keep the system running without interruption as long as sufficient charge remains in the batteries. When power is restored the charger recharges the batteries over a period of several hours. A low voltage disconnect (LVD) device installed between the batteries and the equipment disconnects the batteries if they reach a level of discharge (~30%) that might result in permanent damage to the batteries. One additional benefit of this approach is that the battery chargers used can easily be substituted for solar panels and/or wind turbines for sites that are entirely without grid electricity.

All EMR-related hardware is powered from this centralized power cabinet. TCW appliances and thermal label printers located in adjacent rooms are powered by cables fed out from the top of the cabinet via electrical conduits. The battery charger provides a single point of contact between the mains power and the EMR power backup system. Consequently, it is sufficient for protect for voltage spikes and electrical noise commonly resulting from lightning storms, as well as under and over voltage condition resulting from mains surges or brown-out conditions at just this one location.

While we were able to find -48VDC power supplies from Dell, most manufacturers do not support this voltage. To address this issue we use DC/DC power supplies, which take the 48VDC and convert it down to the voltage required to run the device. In the example of our current server we use 12VDC, and for our touchscreen clinical workstation appliances (TCW) (Text S5) we use 19VDC. Figure S1.B shows an alternate cabinet configuration and employs the current configuration of server. Note the small plastic box mounted to the top of the server cabinet containing a GPRS (General Packet Radio Service) modem used for remote monitoring of the server and power backup system from a central location.

With increased emphasis on reducing carbon footprint in the West, DC power distribution is now becoming the trend for data centers and server farms, where it has been

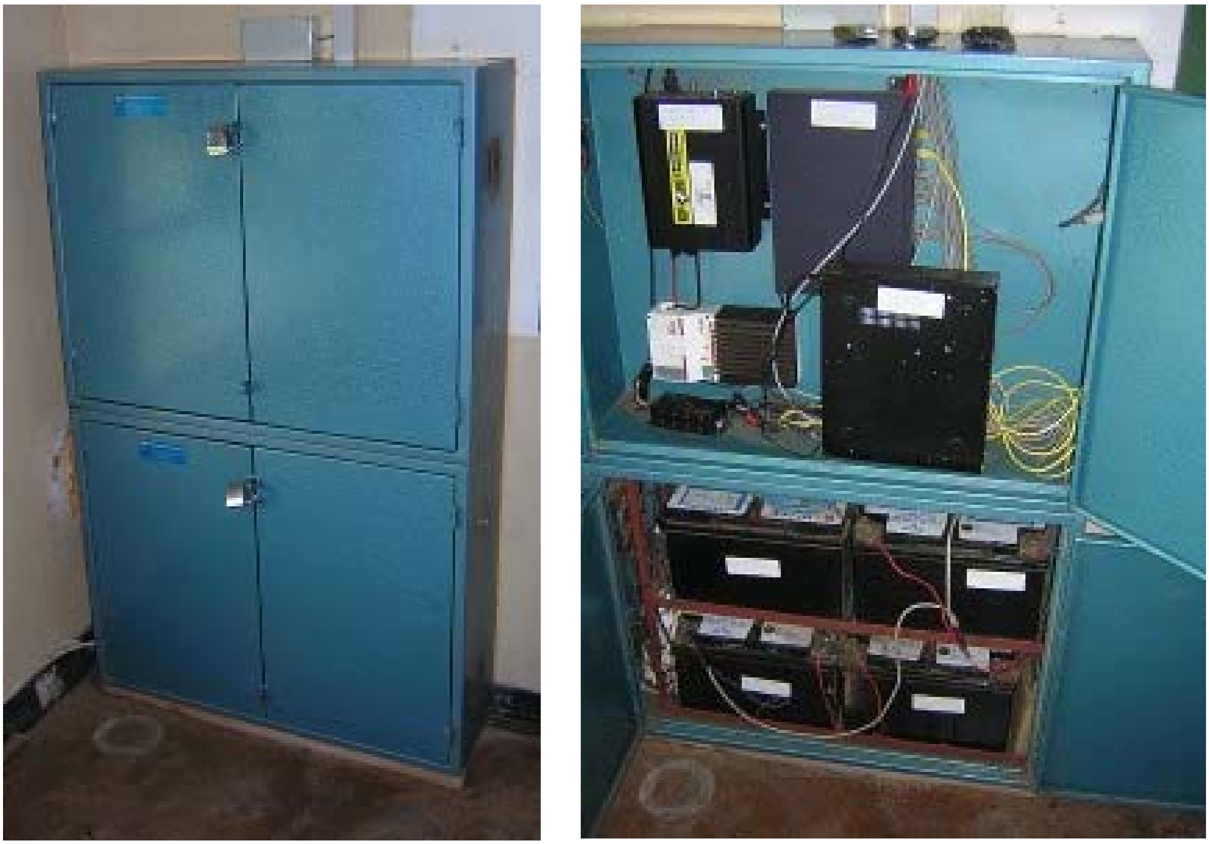

estimated that energy loss in AC to DC conversion alone was roughly 29% [1].

Figure S1.B: Current cabinet configuration with new small-footprint server (Image Credit: Samuel Manda).

Low-Power Computing

In Malawi, the vast majority of care is not delivered in central or district hospitals. Rather it is delivered through the many health centers scattered around the country, a significant number of which are off the main electrical grid. Recognizing that the ultimate goal was to enable EMR systems to operate at these sites, we focused on optimizing our hardware to consume as little power as possible. To pilot our power backup system in this context, we operationalized two health centers in September 2008 on the outskirts of Lilongwe that are off the mains grid using a hybrid solar/wind-powered solution.

Dedza District hospital is one of the ART EMR pilot sites in Malawi. In October 2008 an electrical fire destroyed part of the hospital where the main electrical panel was located. It took two days to restore power to the hospital, during which time no lighting or electrical outlets were functional. Despite this prolonged power outage, the EMR system functioned without interruption, running entirely from the battery backup system. This level of endurance was only possible due to the power considerations during system design.

The ability of the system to perform under these extreme conditions was an important design consideration. While electrical fires are not common, power cuts or load shedding episodes occur frequently, as stated above. Hospitals and health centers are not exempt from these power interruptions.

An additional advantage if using low-power devices is the provision for using Power-over-Ethernet [2]. This allows the TCW appliance to receive its power and data connection using a single data cable., reducing the time and cost required to deploy a workstation.

Using low-power devices we are able to run three touchscreen clinical workstations appliances, complete with thermal label printers, a network switch and a server with the combined power consumption lower than a single 100 Watt light bulb (Figure S1.C).

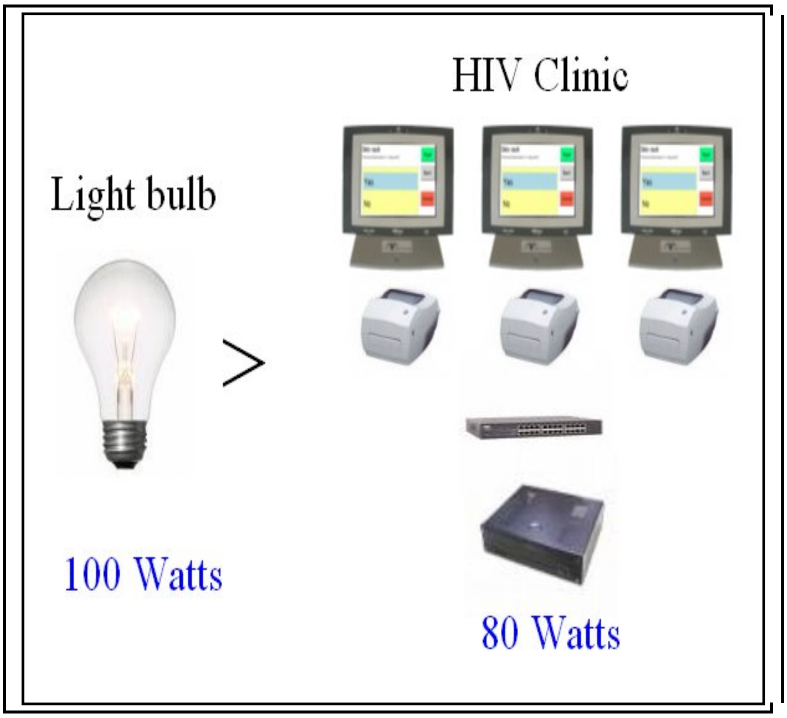

Figure S1.C: A small ART EMR deployment consumes less power than a 100 Watt light bulb

References

1. de Jong ECW and Vaessen PTM. DC power distribution for server farms. Available: http://www.leonardo-energy.org/drupal/book/export/html/2222. Accessed 15 March 2010.

2. Wikipedia. Available: http://en.wikipedia.org/wiki/Power_over_Ethernet. Accesses 15 March 2010.
