# Supplementary material for: Using Touchscreen Electronic Medical Record Systems to Support and Monitor National Scale-Up of Antiretroviral Therapy in Malawi
Source: PLoS Med. 2010 Aug 10;7(8):e1000319. doi: 10.1371/journal.pmed.1000319 (PMC2919419; doi:10.1371/journal.pmed.1000319)
Supplement: Text S2 — Challenges and Solutions in Implementing the EMR in Malawi (0.01 MB PDF) [file pmed.1000319.s002.pdf]

Challenges and solutions for the successful adoption of Point-of-Care (POC) EMR in resource-constrained countries

Deployment Challenge	Solution in ART EMR
Low levels of computer literacy among health professionals present a significant barrier to the successful adoption of an EMR. Having the computer at the POC where the healthcare worker has the task of using the system while concurrently managing the patient adds to the challenge.	For successful adoption of the system it is critical that the system is easy to learn and easy to use. Toward this end, a system based entirely on a touchscreen user interface has been created.
Absence of unique patient identifiers makes it difficult to link the past medical history to the current patient visit, thus compromising continuity of care, which is critical, particularly for the management of chronic illness.	In the absence of unique patient identifiers in Malawi a touchscreen-based system for assigning ID numbers was created. The system produces adhesive barcoded labels to be affixed to patients' health passports (Text S6). To eliminate the stigma of HIV patients' being identified by registration labels, the issuing of ID numbers is hospital-wide. By December 2009 more than 1,100,000 nationally unique patient ID numbers had been issued across the six EMR host sites.
Low staffing levels combined with a high demand for clinical services result in short patient contact times in the clinic. Any additional workload resulting from system use will compromise the successful adoption of the system.	The HIV EMR system ensures that the electronic system replaces the paper-based capturing of information and does not just act as a duplicate system.
To further address the high demand for clinical services, task shifting is often done with lesser-qualified cadres of health care worker performing patient care.	In Malawi, and much of sub-Saharan Africa, ART delivery is commonly task-shifted to clinical officers and nurses [1]. The design of the touchscreen EMR supports use by lower cadre staff (including clerks and patient attendants for patient registration and vital signs recording) by encoding protocols into the system wherever possible, supporting task shifting in the ART clinic.
The traditional paper-based treatment card and register provide transparency with the data. In contrast, the “black box” nature of	To address this problem a set of data cleaning and supervision tools have been created to provide increased visibility in to the data stored in the

the EMR can create a lack of visibility in the system for the users, and a consequent lack of ownership and lack of confidence in the accuracy of the data and resulting reports.	EMR. Increased visibility into the data is also provided through “active” on-screen reports that allow the user to tunnel down to a patient list from any indicator.
The unreliability of existing electrical power, and indeed the absence of power at many health facilities, is a barrier to the use of electronic systems.	The HIV EMR system has been designed around extremely low power appliance-model computer hardware, combined with an innovative power backup system capable of keeping the EMR running for more than 48 hours in the absence of power. A small installation consumes less power than a single 100 Watt light bulb and is capable of being run off a modest solar power system (Text S1).
Computer support systems in resource constrained settings (particularly Africa) are weak and computers often break down due to poor maintenance, viruses, power surges, and misuse.	Touchscreen clinical workstation (TCW) appliances have been optimized for high reliability and maintainability based on the “information appliance” concept (Text S5). The Linux open source operating system is used on both workstations and servers, providing a stable environment that is relatively immune to current computer viruses.

References

1. Zachariah R, Ford N, Philips M, Lynch S, Massaquoi M, et al. (2009) Task shifting in HIV/AIDS: opportunities, challenges and proposed actions for sub-Saharan Africa. Trans R Soc Trop Med Hyg 103: 549-558.
