# Supplementary material for: Using Touchscreen Electronic Medical Record Systems to Support and Monitor National Scale-Up of Antiretroviral Therapy in Malawi
Source: PLoS Med. 2010 Aug 10;7(8):e1000319. doi: 10.1371/journal.pmed.1000319 (PMC2919419; doi:10.1371/journal.pmed.1000319)
Supplement: Text S3 — Commitment to Open-Source Software (0.10 MB PDF) [file pmed.1000319.s003.pdf]

Commitment to Open Source Software

The EMR is built using the same high quality open source operating systems, database management systems and web application servers as Google, Twitter and IBM. These systems are developed and maintained by a community of software developers from all over the world including both unpaid volunteers and employees paid by their companies to work on such systems. The software that results from this community tends to be highly tested, secure and reliable.

Besides quality, open source software is also generally available at no cost. While software licensing costs may be small relative to the cost of hardware, deployment, maintenance and support, these costs are significant when rolling out a system to multiple sites. With open source software there is no licensing cost, so additional deployments incur no additional cost. Furthermore, software updates are always free, although an Internet connection may be required.

Another benefit of using open source software is that its globally distributed nature enables a community based support system. Support channels are often informal but highly accessible in the form of email lists, wikis and Internet relay chat (IRC) channels. These support mechanisms are highly suited to developing countries, where in-country support for any kind of software is typically not available and the cost of international calls to a help desk is prohibitive.

Not only do we use open source software, but we are also active and vocal members of the open source community. We contribute to the community by answering questions in IRC, adding to wikis, and blogging about our experiences. Perhaps most importantly, we release all software that we have developed under an open source license so that others may contribute and benefit from our software (see www.github.com/baobab). Our complete open source software stack is shown below in Figure S3.

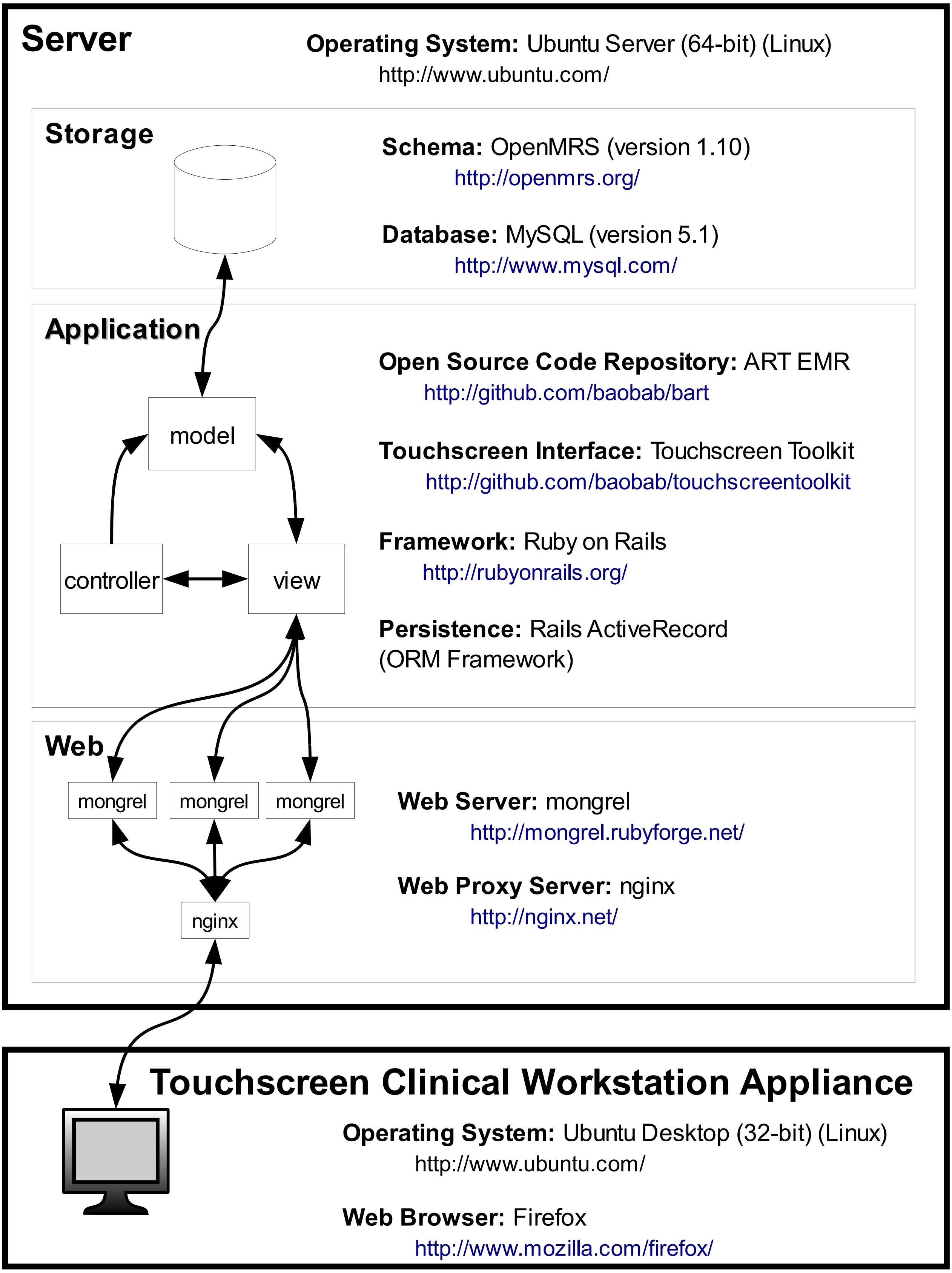

Figure S3: Open source software stack
