# Supplementary material for: Using Touchscreen Electronic Medical Record Systems to Support and Monitor National Scale-Up of Antiretroviral Therapy in Malawi
Source: PLoS Med. 2010 Aug 10;7(8):e1000319. doi: 10.1371/journal.pmed.1000319 (PMC2919419; doi:10.1371/journal.pmed.1000319)
Supplement: Text S4 — Importance of Flexibility to Support Varying Workflows (0.09 MB PDF) [file pmed.1000319.s004.pdf]

Differing Workflows Require Flexible Software

Below we show patient workflow diagrams for three different hospitals. Each site has differences in how they manage patients.

At the Lighthouse clinic (Figure S4.A) patients are triaged at a nursing station for referral to a doctor or clinical officer. However, at Salima district hospital (Figure S4.B) the clinical officer on duty is only available in the ART clinic in the mornings. Consequently patients are triaged at reception to ensure that all patients requiring a consultation with the clinical officer are identified as early in the day as possible. Zomba central hospital (Figure S4.C) has a separate workflow for Pre-ART patients (HIV-positive patients who are currently ineligible for receiving ART).

The ART EMR is designed to be sufficiently flexible to accommodate different workflows. This is implemented using a set of rules that control what functions of the EMR may be accessed based on the location of the touchscreen clinical workstation (TCW) appliance.

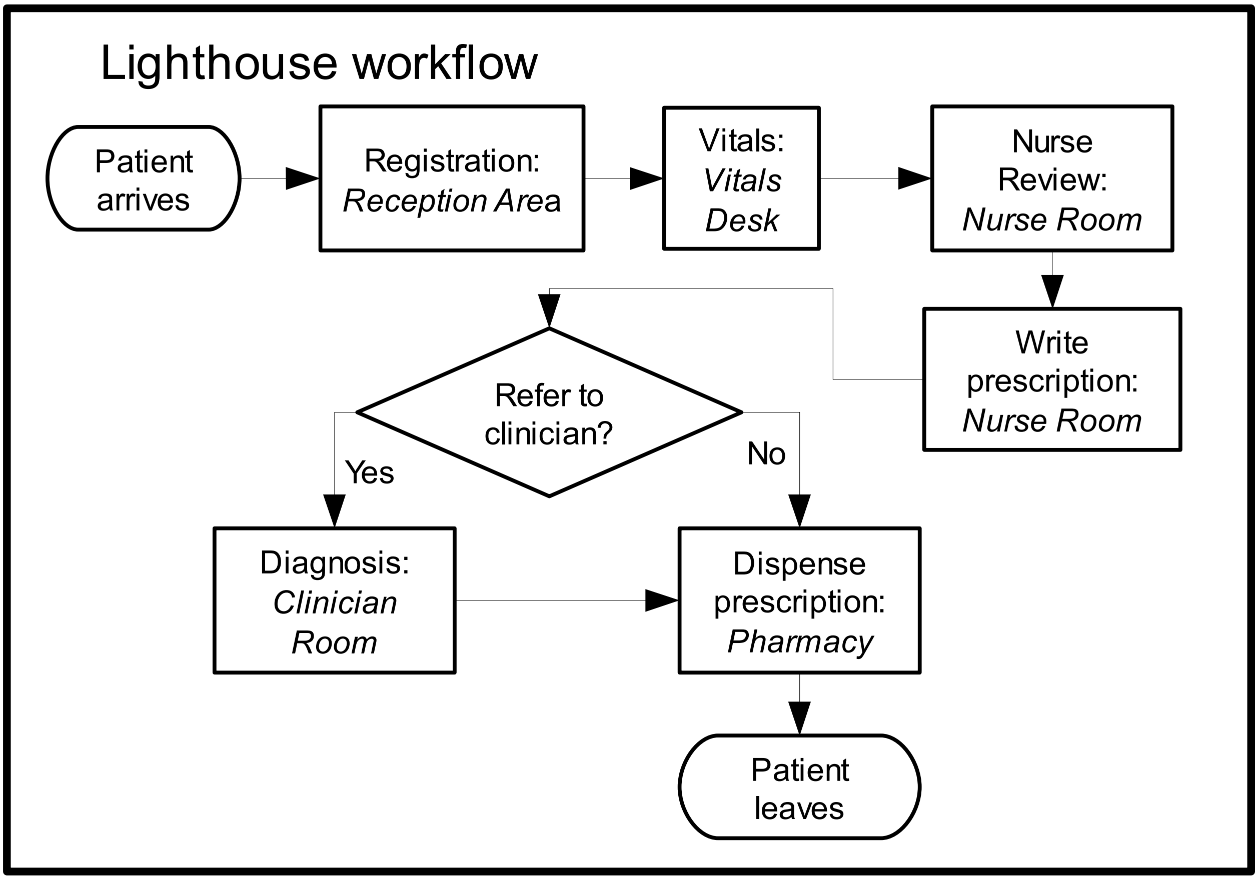

Figure S4.A: Lighthouse clinic workflow diagram

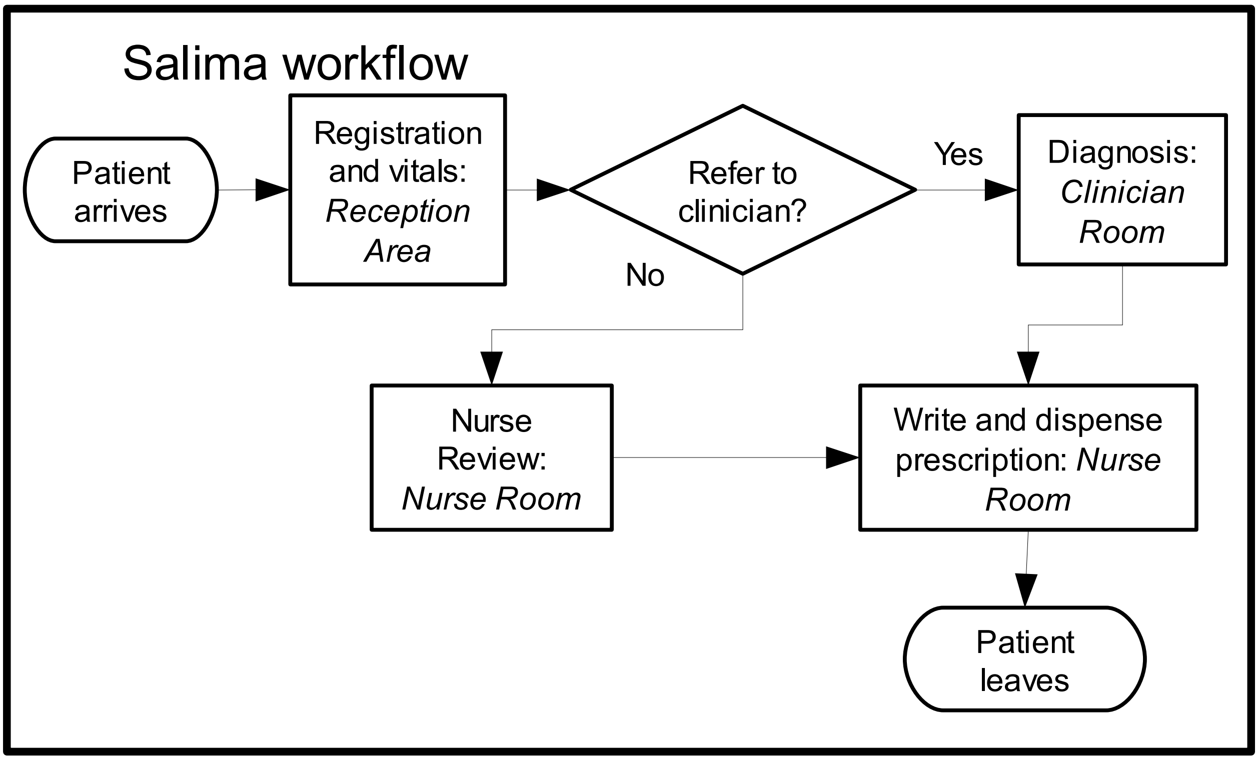

Figure S4.B: Salima district hospital workflow diagram

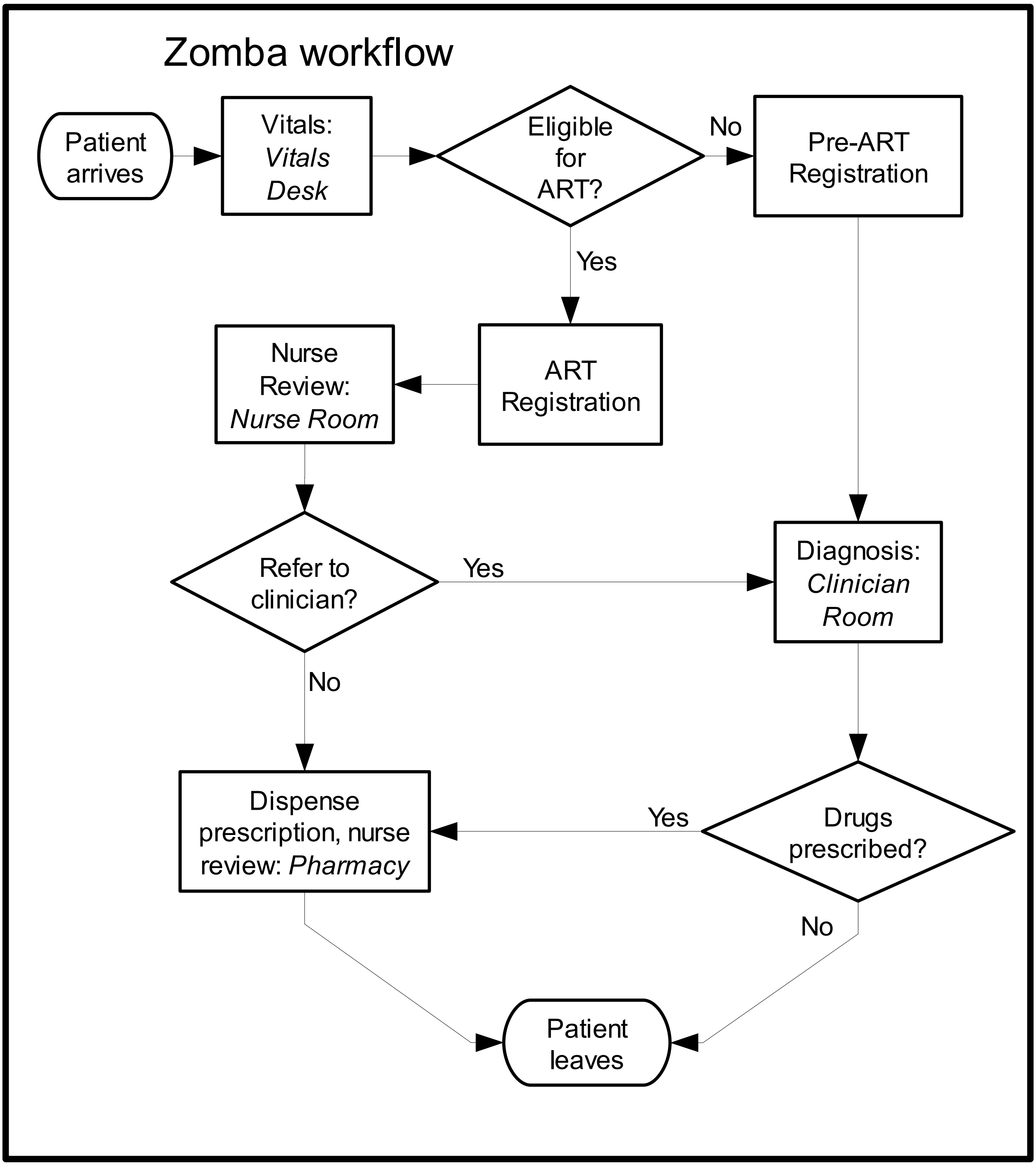

Figure S4.C: Zomba central hospital workflow diagram
