# Supplementary material for: Using Touchscreen Electronic Medical Record Systems to Support and Monitor National Scale-Up of Antiretroviral Therapy in Malawi
Source: PLoS Med. 2010 Aug 10;7(8):e1000319. doi: 10.1371/journal.pmed.1000319 (PMC2919419; doi:10.1371/journal.pmed.1000319)
Supplement: Text S5 — The Touchscreen Clinical Workstation Appliance (0.06 MB DOC) [file pmed.1000319.s005.doc]

**The Touchscreen Clinical Workstation (TCW) Appliance**

**Appliance-Model Computing**

Norman introduces the notion of an information appliance in his book “The Invisible Computer: Why Good Products Can Fail, the Personal Computer Is So Complex, and Information Appliances Are the Solution” [1]. The notion of “invisible” here tries to convey the idea of the computer disappearing. This happens when the computer is no longer perceived to be one. Desktop computers are designed to address multiple needs making them the “jack-of-all-trades” but master of none. The appliance model has a number of benefits. Appliances are optimized for one particular task: they contain only the necessary components to achieve the specific goal. Consequently they are less expensive to manufacture. Furthermore their form and function is optimized for usability. Many devices that we use in everyday life are appliance model computers. Examples include the TIVO (digital television recorder) and the iphone.


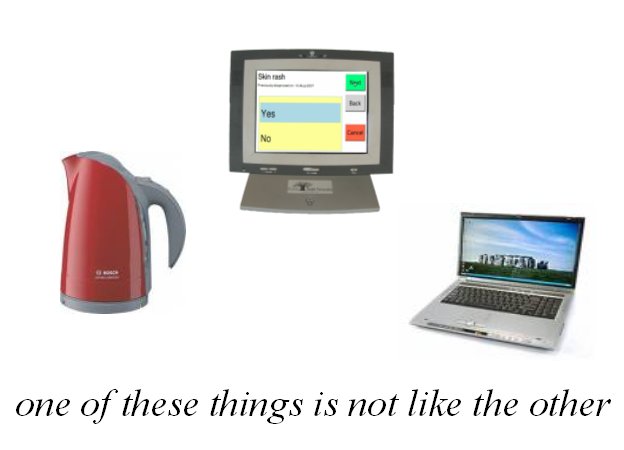
Figure S5: The TCW is more similar to the electric kettle than to the laptop

We designed the TCW as an appliance model computer, with the sole purpose of running the clinical application. When the device starts up, the user is taken directly to a web browser running in full-screen mode and a default web page is loaded showing the login screen to the clinical application. We based our first TCW appliance on an Internet appliance called a Netpliance I-Opener, designed to provide customers with low-cost Internet access without having to incur the expense of a fully functional computer [2]. Figure S5 shows a clinical touchscreen workstation top and center. Here we challenge the perception that the workstation is a computer by asserting that it is more akin to a kitchen appliance (electric kettle) than a laptop computer.

Our TCW appliance was first conceived during the initial pilot work in 2000 and has been refined over the years. It is based on the notion of a clinical workstation introduced by Safran et al. [3,4]. We introduced hardware features into the design of the device to optimize it for reliability, availability, serviceability, usability and installability in a developing-world setting.

**Contrasting the Applicability of General-Purpose Desktop Computers vs. Special-Purpose Clinical Workstations in the Malawi Healthcare Setting**

Background

Conventional desktop computers have been in use in Malawi for sometime in a variety of settings and with various degrees of success. Below we present a systematic comparison of desktop computers vs. dedicated single-purpose computers such as the TCW appliance.

Hardware Reliability

While the main components of a desktop computer are solid state (no moving parts), many of the peripheral components are mechanical in nature, and consequently subject to mechanical failure. This includes first and foremost the computer’s hard disk drive where programs and data reside. Generally speaking, the hard drive is a fragile device that can be easily damaged by relatively minor physical shock. Two peripherals that commonly fail are the mouse and keyboard. Finally, power supplies and microprocessors overheat and fail as a result of broken cooling fans, often due to excessive build-up of dust that prevents the fan from turning. The failure of any one of these devices can render the computer inoperable. Limited access to repair facilities can cause delays in using the computer resulting in overdue reporting of data and, frequently, loss of data when the hard disk drive fails.

The TCW appliance was engineered specifically to mitigate problems resulting from mechanical failure. The hard disk drive is replaced with a solid-state disk (SSD) device. The touchscreen replaces the mechanical mouse and keyboard as an input device. Finally, the TCW appliance uses a heat sink for cooling, making fans unnecessary.

Hardware Maintainability

When a desktop computer breaks it must be sent for repair. This process can be both expensive and time-consuming. A vehicle is generally required to get the computer to and from a repair facility. The number of reputable and qualified repair facilities within Malawi is extremely small. Furthermore, there is very limited capacity within the government to maintain its own equipment. Warranties are generally limited and hard to take advantage of.

The TCW appliance is small and can be more easily transported or shipped without significant risk of damage occurring. We have created an infrastructure for repairing and maintaining the TCW appliance within Malawi.

Functionality

Conventional desktop computers can be used to run a variety of office productivity software applications such as word processing, spreadsheets, etc. They can also be used for browsing the Internet, sending emails, playing games, listening to music and watching DVDs. This can be both a strength and a weakness.

The TCW appliance is designed to run a single-purpose piece of software specifically designed to achieve a particular data collection or patient management function. The software is configured in a way that prevents users from gaining access to the operating system and installing their own software on the computer, protecting against the introduction of viruses.

Usability

The conventional keyboard and mouse used on most desktop computers is highly suitable for word processing and similar applications for users with some level of computer experience.

The TCW appliance uses a touchscreen user interface to accommodate users with little or no computer experience.

Risk of Theft

Desktop computers are clearly recognized as valuable and are consequently at risk of theft. The theft of a computer also results in loss of data stored on it, which can cause delays and additional costs in reporting data.

The TCW appliance cannot easily be used for general computing functions and we believe is consequently less likely to be stolen as it would have little resale value. Based on the absence of the mouse and keyboard, many people do not even recognize it as a computer. The small size of the workstation makes it an easier target for theft. However, fixing the base of the workstation to the desk, or mounting it to a wall for added security can help protect the equipment.

Total Cost of Ownership & Opportunity Cost

The cost of a computer extends far beyond the initial purchase price. Consideration needs to be given to shipping costs, installation costs, life expectancy, and anticipated repair costs over the life of the equipment. This concept is called the total cost of ownership (TCO). Losses, and benefits-not-realized as a result of inoperable equipment should also be taken into account. This might include loss of data that would require resources to re-enter, or delayed reports resulting in loss of funding support, etc.

Anecdotally, we have observed that desktop computers typically last less than three years in Malawi, and we have observed data loss due to computer failure on many occasions. In contrast, many of the TCW appliances at Kamuzu Central Hospital, Lilongwe, Malawi, were installed in early 2001 and have been working without need of repair, or with minor repairs, since they were initially installed. Since no data is actually stored on the workstation there is no risk of loss of data if a workstation is damaged.

Summary

We believe that generally higher reliability combined with lower total cost of ownership of the TWC appliance presents a compelling argument in favor of using dedicated, single-purpose computers where a robust and reliable solution is needed.

References

1. Norman DA (1999) The Invisible Computer: Why Good Products Can Fail, the Personal Computer Is So Complex, and Information Appliances Are the Solution: The MIT Press.
2. Wikipedia. Available: <http://en.wikipedia.org/wiki/I-Opener>. Accessed 15 March 2010.
3. Safran C (1993) Defining clinical workstation. MD Comput. May-Jun;10: 145-6, 92
4. Safran C (1994) Defining clinical 'workstation'. Int J Biomed Comput. Jan; 34: 261-5
