# Supplementary material for: Using Touchscreen Electronic Medical Record Systems to Support and Monitor National Scale-Up of Antiretroviral Therapy in Malawi
Source: PLoS Med. 2010 Aug 10;7(8):e1000319. doi: 10.1371/journal.pmed.1000319 (PMC2919419; doi:10.1371/journal.pmed.1000319)
Supplement: Text S6 — The Malawi Health Passport (0.12 MB PDF) [file pmed.1000319.s006.pdf]

The Malawi Health Passport

The Malawi Health Passport is a small booklet issued by the Ministry of Health, and used as a patient-kept portable medical record. Patients take it with them when they visit a healthcare provider, who in turn write details of the visit in the passport.

The cover of the booklet has a place for the patient's demographic details to be hand-written. Using a touchscreen clinical workstation (TCW) appliance (Text S5) at a centrally-located patient registration window, clerks enter the patient's details directly into the EMR, which then generates an adhesive label showing these details. The EMR assigns the patient a nationally unique patient ID number that is also printed on the label along with its barcode representation, complete with check digit. Figure S6 shows a health passport with a ID label attached. A short video showing the patient registration process can be viewed at http://www.youtube.com/watch?v=mTloTU7Az_E.

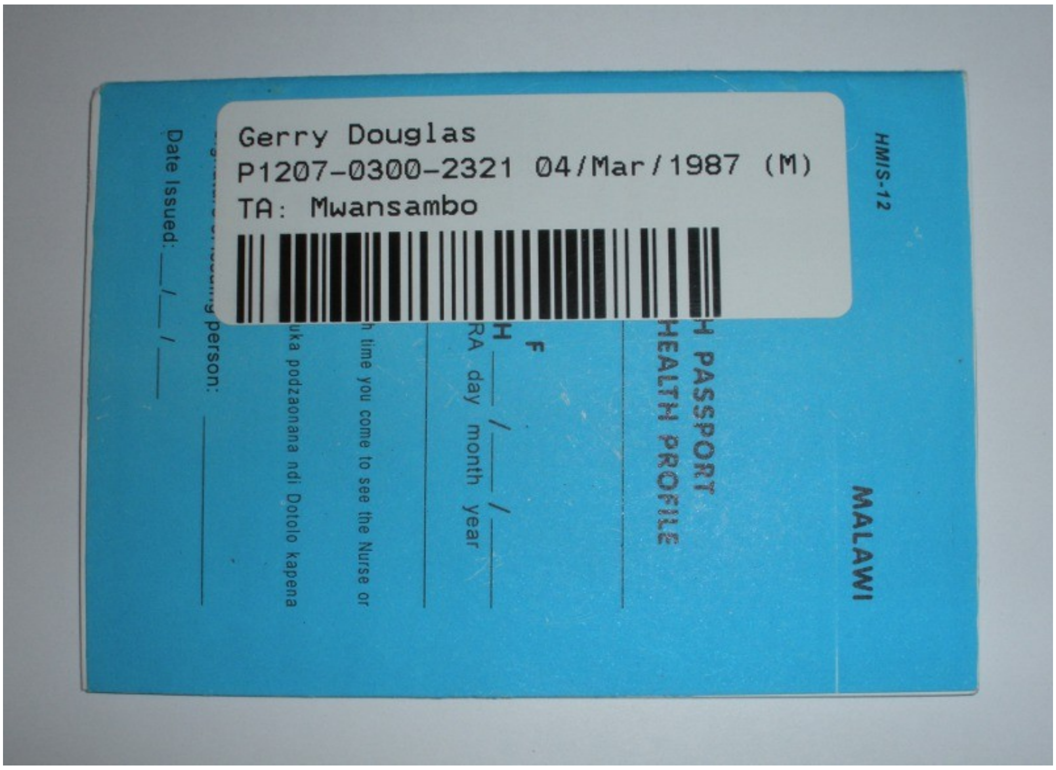

Figure S6: Patient's health passport with ID label attached (Image Credit: Gerald P. Douglas).

Initially we affixed the label horizontally. However, we observed that patients tended to fold their passport lengthwise, parallel to the spine. This resulted in a crease in the barcode, making it more difficult to scan correctly.
