# Supplementary material for: Using Touchscreen Electronic Medical Record Systems to Support and Monitor National Scale-Up of Antiretroviral Therapy in Malawi
Source: PLoS Med. 2010 Aug 10;7(8):e1000319. doi: 10.1371/journal.pmed.1000319 (PMC2919419; doi:10.1371/journal.pmed.1000319)
Supplement: Text S7 — The Touchscreen Toolkit (0.53 MB PDF) [file pmed.1000319.s007.pdf]

The Touchscreen Toolkit

We created the Touchscreen Toolkit to simplify the development of touch-friendly user interfaces without requiring each page to be individually and consistently customized for touchscreens. It is a Javascript library that can be added to any HTML form. The library can be downloaded from http://github.com/baobab/touchscreentoolkit.

The Touchscreen Toolkit transforms standard HTML forms (Figure S7.A), just after they are loaded in a browser, into a set of wizard-like screens (Figures S7:B-E) showing each of the input fields on separate screens.

For each input field, the toolkit displays a customized full-screen window with:

- Appropriate input controls
- User instructions
- Global navigation buttons (Next/Back)
- Data validation criteria (optional)

Fields can be “skipped” if a specified condition for each of the fields is not met.

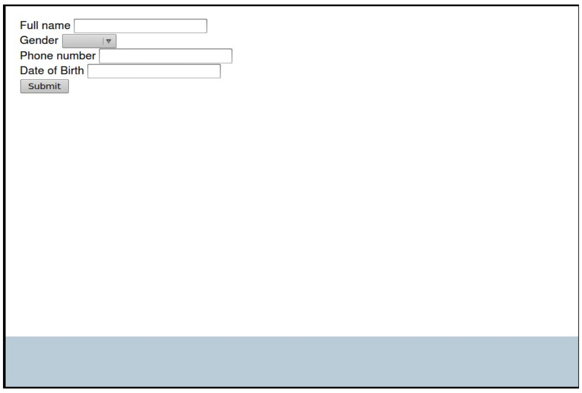

Figure S7.A: Original HTML Form

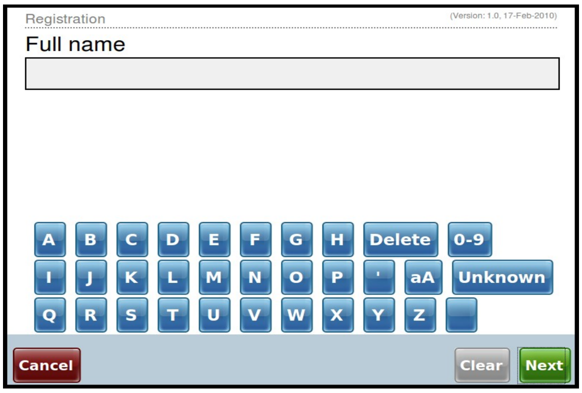

Figure S7.B: First wizard screen

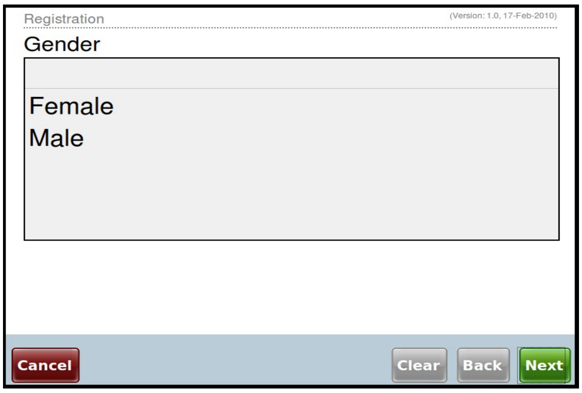

Figure S7.C: Second wizard screen

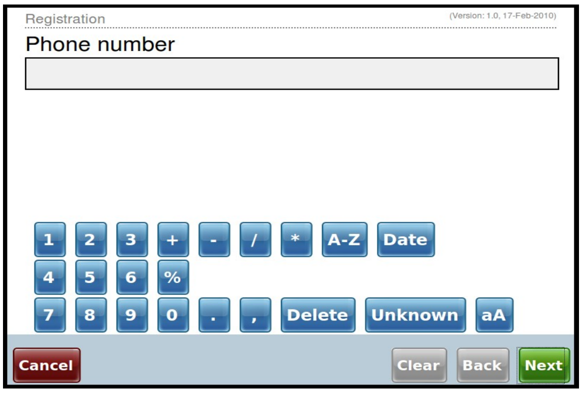

Figure S7.D: Third wizard screen

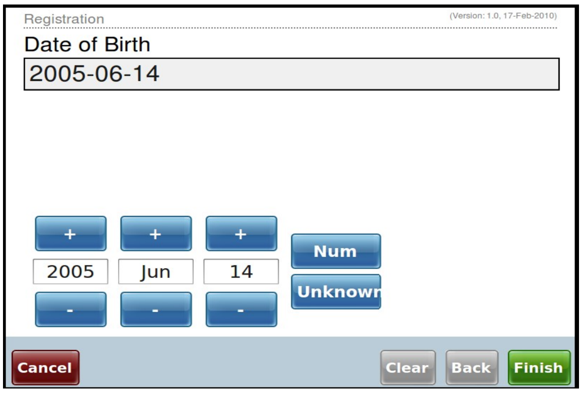

Figure S7.E: Final screen (note “Finish”)
