# Supplementary material for: Using Touchscreen Electronic Medical Record Systems to Support and Monitor National Scale-Up of Antiretroviral Therapy in Malawi
Source: PLoS Med. 2010 Aug 10;7(8):e1000319. doi: 10.1371/journal.pmed.1000319 (PMC2919419; doi:10.1371/journal.pmed.1000319)
Supplement: Text S8 — Scalability, Localization, and Customization of the ART EMR (0.01 MB PDF) [file pmed.1000319.s008.pdf]

Scalability, Localization and Customization of the ART EMR

Scalability was engineered into the design of the system. The hardware platform is designed for the most resource-constrained of settings such as a small health center, off the electrical grid, and staffed by a nurse and a clerk. The system is built on free and open source enterprise architecture software (Text S3). Early versions of the hardware was highly customized to meet specific needs. However, current deployment use off-the-shelf hardware.

However, the application software is highly tailored to Malawi's approach to undertaking, monitoring and evaluating healthcare delivery. The user interface is in English, and currently does not lend itself to use in other languages. The system uses an older version of the OpenMRS data model [1], but this is being updated in the next version, scheduled to be released in July 2010.

The system is not highly customizable, and would require a moderate level of proficiently with the programming language in which the application software is developed (Ruby on Rails) as well as an understanding of the OpenMRS database schema (Text S3).

References

1. Waters E, Rafter J, Maganga RT, Fraser HSF, Douglas GP, et al. (2009) Integration of a Touchscreen System for Patient Registration and Primary Care Data Collection with OpenMRS at Neno District Hospital, Malawi. AMIA Annu Symp Proc 107.
