# Supplementary material for: Using Touchscreen Electronic Medical Record Systems to Support and Monitor National Scale-Up of Antiretroviral Therapy in Malawi
Source: PLoS Med. 2010 Aug 10;7(8):e1000319. doi: 10.1371/journal.pmed.1000319 (PMC2919419; doi:10.1371/journal.pmed.1000319)
Supplement: Text S9 — Benefits of the Point-of-Care Model (0.01 MB PDF) [file pmed.1000319.s009.pdf]

Benefits of the Point-of-Care (POC) Model

The use of electronic medical record (EMR) systems can provide benefits to healthcare delivery. The point-of-care (POC) approach represents the highest level of interaction between the healthcare worker (HCW) and the information system since it (generally) requires that the interaction take place during the clinical encounter. While this is challenging to achieve, it offers the greatest benefits. The POC approach has benefits for the HCW using the system, the patients receiving care, and those responsible for monitoring and evaluation (M&E). We describe seven benefits here in the context of the antiretroviral (ART) EMR system.

1. Improvements in data completeness and accuracy, and timeliness of reports

1) Data entry is done by the HCW who collect the data and understand its meaning, and this potentially contributes to improved data quality.
2) Errors and inconsistencies in data identified through computerized validation rules can be more easily corrected while the patient is present (Text S10).
3) There is never a backlog in data entry, so reports can be run at any time.

2. Clinical decision support

1) The HCW receives alerts (e.g. patient body mass index (BMI) <18 – start nutritional support)
2) The HCW receives reminders (e.g. >6 months since previous CD4 lymphocyte count)

3. Encoding of clinical protocols and guidelines in the system

1) The EMR helps the HCW to follow a standard treatment protocol that includes review of side effects, drug adherence, ART regimen, dosage and use of cotrimoxazole

prophylaxis, resulting in a more standardized and comprehensive visit for each patient.

2) The HCW's adherence to using protocols may be measured using the data collected by the system (data in the system is linked to a particular HCW by system User ID and login details), and through feedback to the HCW adherence to using protocols can be improved [1].
3) Patients are expected to have better outcomes if protocols are adhered to.

4. Improvements in efficiency

1) Having the data in electronic form at POC means that they can be reproduced to meet documentation needs at the POC (a form of "highly adaptive carbon paper"). For example, a newly-established diagnosis may need to be documented in the patient's health passport (Text S6) and/or on a discharge form, in a ward register, on a multi-part prescription. Significant improvement in efficiency is achieved by capturing this information once only, and printing it out in context-applicable ways onto inexpensive adhesive labels.
2) Retrieval of individual patient records at clinic level is rapid through barcode identification, and is not subject to missing paper files.

5. Immediate availability of information in the system to the HCW while managing the patient

1) The HCW has access to current (e.g. CD4 count result from earlier in the day) and historical (e.g. past medical history) clinical data, presented in a systematic way through a patient "dashboard".

6. Supporting a team-based approach to patient management

1) Multiple touchscreen clinical workstation (TCW) appliances (Text S5) allow immediate sharing of patient information between different locations in a clinic during a patient visit (e.g. patient registration, vital signs station, nurse examination room, doctor examination room, pharmacy).

7. Clinical calculation

1) Body mass index (BMI) calculation based on patient's weight and height.
2) Dosing of paediatric antiretroviral drugs based on child's weight.
3) Adherence to taking medication based on last and current visit date, pills remaining and pills dispensed at last visit.

References

1. Landis Lewis Z, Mello-Thoms C, Visweswaran S, Crowley R (2010) Using electronic medical records to measure guideline adherence in low-resource settings. Medinfo 2010. In Press.
