# Supplementary material for: Using Touchscreen Electronic Medical Record Systems to Support and Monitor National Scale-Up of Antiretroviral Therapy in Malawi
Source: PLoS Med. 2010 Aug 10;7(8):e1000319. doi: 10.1371/journal.pmed.1000319 (PMC2919419; doi:10.1371/journal.pmed.1000319)
Supplement: Text S10 — Dynamic and Two-Level Data Validation (0.07 MB PDF) [file pmed.1000319.s010.pdf]

Dynamic and Two-Level Data Validation

The ability to perform data validation during the time of entry can significantly improve data quality. Traditional approaches to validation use range checking. Upper and lower limits are set and data outside the range is disallowed.

Increased accuracy can be achieved using dynamic range checking. Here, the upper and lower limits are not fixed. Rather the limits move dynamically based on a rule or set of rules. For example, in Figure S10 below you can see a “Value out of Range” warning indicating the valid range for the patient’s weight is between 28 and 76 Kg. This range is calculated using the patient’s date of birth and a look-up table of weights for upper and lower limits for a patient of a specific age to the closest year.

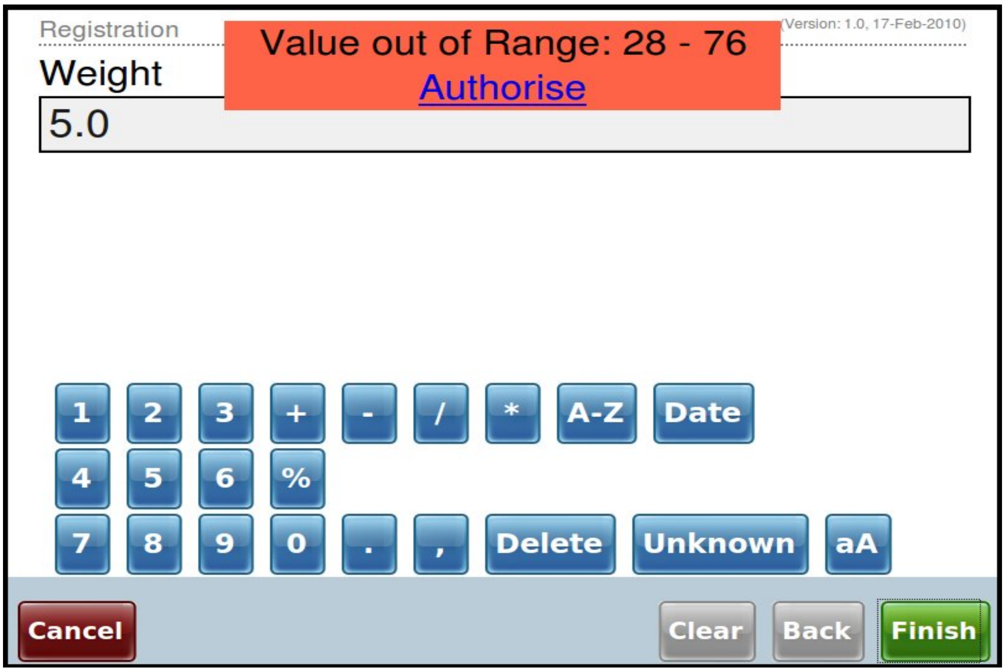

Figure S10: Screen showing out of range error message

A major limitation of this approach to range checking is that if it is too constrained then the system may actually prevent the user from entering an extreme but valid data value. To get around this problem we have implemented two-level range checking. Here we create two ranges. The first range traps values in the traditional way, preventing unfeasible values from being entered. The second range traps values that are unlikely but plausible if they were

valid outliers. This technique slows the user down, forcing them to look closely at what they have entered. If the user recognizes they have made a transposition error for example, typing 27 instead of 72, then they can enter the correct value. However, if the value is truly 27, then the user can authorize the unlikely but correct value by entering their password, allowing the data to be saved. We believe the process of slowing the user down and forcing them to take a second look at the data value improves data accuracy.
